# Magnetic Interactions in Ferrite Bead-Enhanced Wiegand Wires Evaluated by First-Order Reversal Curves

**DOI:** 10.3390/ma18194477

**Published:** 2025-09-25

**Authors:** Chao Yang, Liansong Guo, Guorong Sha, Liang Jiang, Zenglu Song, Yasushi Takemura

**Affiliations:** 1 School of Electrical Engineering, Nanjing University of Industry Technology, 1 Yangshan North Road, Nanjing 210023, China; yangchao@niit.edu.cn (C.Y.); shaguorong@niit.edu.cn (G.S.); jiangliang@niit.edu.cn (L.J.); zenglu_song@niit.edu.cn (Z.S.); 2 Department of Electrical and Computer Engineering, Yokohama National University, Yokohama 240-8501, Japan; takemura-yasushi-nx@ynu.ac.jp

**Keywords:** wiegand wire, first-order reversal curve (FORC), magnetization reversal, large Barkhausen jump, ferrite beads

## Abstract

Wiegand sensors are essential components in self-powered Internet of Things (IoT) nodes, as they can output pulse voltages without an external power supply. Previous research has established that the attachment of ferrite beads to Wiegand wire terminals substantially enhances the sensor’s pulse voltage output. However, the fundamental mechanism responsible for this enhancement remains unclear at the microscopic magnetic level. This investigation systematically examines how ferrite bead attachments alter magnetization reversal processes, Barkhausen jump characteristics, and the energy output in Wiegand wires. Experimental results reveal that ferrite beads enhance irreversible magnetization, modify interaction distributions, and transform the magnetic structure of Wiegand wires. These modifications collectively result in a 1.5–2.0 times higher pulse voltage amplitude and 30–40% greater output energy, establishing a theoretical framework for Wiegand sensor optimization. The research methodology combines vibrating sample magnetometer (VSM) measurements with first-order reversal curve (FORC) analysis to elucidate the underlying micromagnetic mechanisms.

## 1. Introduction

With the rapid development of Internet of Things (IoT) technology, wireless monitoring nodes are increasingly utilized in fields such as industry, agriculture, and healthcare [1]. However, traditional wireless sensor nodes rely on battery power, and due to their finite operational lifespan, regular battery replacement incurs high maintenance costs. This significantly impedes the large-scale deployment of IoT systems. Self-powered sensors enable an autonomous power supply by harvesting ambient energy sources (e.g., magnetic energy, vibration energy), thereby offering an effective solution to this challenge. Among these, Wiegand sensors have garnered significant research attention owing to their unique magnetic characteristics [2,3].

Wiegand sensors operate via the Wiegand effect, whose core component is the Wiegand wire (typically with a composition of Fe_0.4_Co_0.5_V_0.1_). This wire is composed of a soft magnetic outer layer and a hard magnetic core [4,5]. When the polarity of the external magnetic field reverses, the soft magnetic layer undergoes a rapid magnetization reversal, accompanied by a large Barkhausen jump, thus generating a pulse voltage in the surrounding pickup coil [6]. Compared with Hall sensors, electromagnetic sensors, and other counterparts, the pulse amplitude and width of Wiegand sensors are independent of the rate of magnetic field variation [7]. Additionally, they can generate energy even at extremely low magnet motion speeds (with a single-pulse energy of approximately 600 nJ) [6], thus offering unique advantages for powering low-power devices.

To meet the power consumption demands of wireless nodes, enhancing the output energy of Wiegand sensors is critical. Previous studies have demonstrated that ferrite bead attachments at Wiegand wire terminals enhance the pulse voltage amplitude by 1.5–2.0 times and output energy by 30–40% [8]. However, the intrinsic mechanism underlying this energy enhancement mechanism has not been elucidated via the analysis of microscopic magnetic properties. Traditional hysteresis loops [4] characterize macroscopic magnetic properties, yet they fail to reveal either the interfacial interaction between the soft magnetic layer and the hard magnetic core or the detailed process of magnetization reversal.

As an advanced magnetic characterization technique, first-order reversal curves (FORCs) enable the characterization of a material’s internal coercivity distribution, interlayer interactions, and multiphase structure by analyzing the distribution of irreversible components during magnetization reversal [9,10]. For example, FORC diagrams can distinguish between the rapid reversal of the soft magnetic layer and the slow reversal of the hard magnetic core within the Wiegand wire, while simultaneously quantifying the magnetostatic coupling between them [11,12]. The combination of Vibrating Sample Magnetometer (VSM) [13,14] measurements and FORC diagrams analysis provides a comprehensive approach to elucidate the internal mechanism by which ferrite beads enhance sensor output, from the perspectives of magnetic flux distribution and interlayer interaction [13].

A Vibrating Sample Magnetometer (VSM) was utilized to characterize the hysteresis loops and FORCs of Wiegand wires both with and without ferrite beads. By combining these measurements with the quantitative analysis of FORC diagrams [15,16], the effects were determined for ferrite beads on the internal magnetization intensity distribution of Wiegand wires and on the interaction between the soft layer and the hard core. These analyses elucidated the intrinsic reason underlying ferrite beads’ enhancement of sensor output, thereby establishing theoretical support for the performance optimization of Wiegand sensors. Experimental results demonstrated that following the attachment of ferrite beads, the saturation magnetization of the Wiegand wire decreased, while the amplitude of the Barkhausen jump in the minor loop increased. Further analysis of the FORC diagrams revealed that the placement of ferrite beads at both ends of the Wiegand wire induces three significant modifications: the enhancement of irreversible magnetization, modification of the interaction distribution, and the transformation of magnetic structure. These structural modifications facilitate an increased volume of irreversible magnetic reversal within the soft magnetic layer of the Wiegand wire, consequently enhancing the amplitude and energy of the pulse voltage induced in the coil. This study offers a theoretical foundation based on microscopic magnetic mechanisms for the optimization of Wiegand sensor performance.

## 2. Materials and Methods

**Figure 1:**
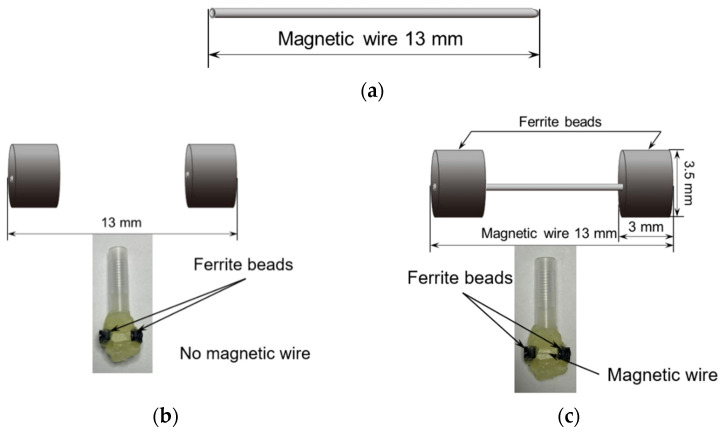
Experimental configurations of Wiegand wire: (**a**) “only wire” model; (**b**) “only beads” model; and (**c**) “affected wire” model.

In this study, a Wiegand wire (13 mm in length, 0.25 mm in diameter) with a composition of Fe_0.4_Co_0.5_V_0.1_ was employed. The wire was supplied by SWFE Co., Ltd. (Meishan, China), as shown in Figure 1a. Detailed specifications of the sample are available in other literature [6,8].

This Wiegand wire possesses a dual-layer structure: a soft magnetic outer layer and a hard magnetic core. It is fabricated through a torsion process, where the outer layer demonstrates lower coercivity than the core—with coercivity values of approximately $\mu_{0}H$ = 2 mT and 8 mT, respectively ($\mu_{0}$ denotes the magnetic permeability in a vacuum) [16,17]. As discussed previously [6], the coercive force exhibits gradual variation along the radial direction of the wire.

The ferrite beads (TDK, Co. Ltd., Tokyo, Japan, HF57BB3.5X3X1.3) possess an inner diameter of 1.3 mm, an outer diameter of 3.5 mm, and a length of 3 mm, comprising nickel, zinc, and iron as their primary metallic components [6]. Figure 1b depicts the ferrite beads in the model without the Wiegand wire, which is designated as the “only beads” model. To investigate the influence of ferrite beads on the Wiegand wire, ferrite beads were attached to both ends of the wire in the current investigation, as shown in Figure 1c; this configuration is designated as the “affected wire” model. Correspondingly, the configuration shown in Figure 1a is designated as the “only wire” model throughout this study.

A Vibrating Sample Magnetometer (VSM) serves as a principal instrument for characterizing the magnetic properties of materials [18]. This technique permits the quantification of various fundamental magnetic properties of magnetic materials, including magnetization curves, hysteresis loops, and demagnetization curves. Since major and minor hysteresis loops are insufficient for characterizing critical features such as magnetic interactions or coercivity distributions, FORCs and FORC diagrams were utilized [12,19]. This approach enables a more comprehensive investigation of Wiegand wires with ferrite beads, including the analysis of magnetic interactions, coercivity distributions, and multiphase identification through a VSM (Model 8600 Series, Lake Shore Cryotronics, Inc., Westerville, OH, USA).

The FORC measurement protocol was implemented according to the following procedure: first, the Wiegand wire was magnetically saturated in a large positive magnetic field ($\mu_{0}H_{\mathrm{sat}}$). The magnetic field was then reduced to a predefined reversal field ($\mu_{0}H_{a}$). A single FORC is defined as the magnetization curve obtained by incrementally increasing $\mu_{0}H_{a}$ back to $\mu_{0}H_{\mathrm{sat}}$ in fixed field steps. By repeating this process across a range of $\mu_{0}H_{a}$ values, a series of FORCs was acquired [11,20].

For any FORC, the magnetization corresponding to a given applied magnetic field ($\mu_{0}H_{b}$) is expressed as $M\left(\mu_{0}H_{a},\mu_{0}H_{b}\right)$, where $\mu_{0}H_{b}\geq \mu_{0}H_{a}$. The FORC distribution is mathematically defined by the following mixed second derivative [21]:

(1) $$\rho \left(\mu_{0}H_{a},\mu_{0}H_{b}\right)=-\frac{1}{2}\frac{\partial^{2}M\left(\mu_{0}H_{a},\mu_{0}H_{b}\right)}{\partial \mu_{0}H_{a}\partial \mu_{0}H_{b}}$$

The FORC distribution derived from Equation (1) is visualized through a contour plot. This plot characterizes the onset of hysteresis, specifically the irreversible change in the magnetic moment relative to the initial magnetic state.

To analyze the magnetic properties of the Wiegand wire, a coordinate transformation was implemented, transforming the original $\left(\mu_{0}H_{a},\mu_{0}H_{b}\right)$ coordinate system to the $\left(\mu_{0}H_{c},\mu_{0}H_{u}\right)$ system. The transformation formulas can be expressed as follows:

(2) $$\mu_{0}H_{c}=\frac{\left(\mu_{0}H_{b}-\mu_{0}H_{a}\right)}{2}$$

(3) $$\mu_{0}H_{u}=\frac{\left(\mu_{0}H_{b}+\mu_{0}H_{a}\right)}{2}$$

## 3. Results

### 3.1. Hysteresis Loops

**Figure 2:**
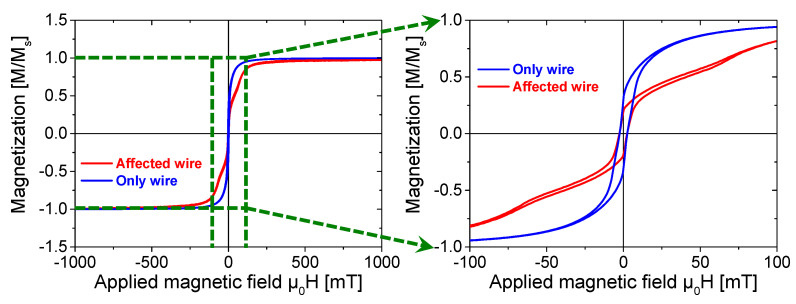
Measured major hysteresis loops of the Wiegand wire under applied fields up to ±500 mT.

Hysteresis loops serve as a fundamental tool for characterizing the magnetic properties of magnetic materials. To extract comprehensive insights into the magnetic performance from hysteresis loops, major hysteresis loops were measured for both the “affected wire” model and the “only wire” model, as presented in Figure 2.

As shown in Figure 2, the two major loops demonstrate nearly identical widths, while the saturation magnetic induction intensity of the “affected wire” model is significantly reduced. The influence of ferrite beads on the Wiegand wire is also evident in the residual magnetic induction intensity and the magnetic permeability of magnetic induction lines. After attaching ferrite beads, the remanence of the Wiegand wire exhibits a significant reduction. Additionally, the rate at which the Wiegand wire reverts to a saturation magnetic induction intensity demonstrates a reduced rate.

**Figure 3:**
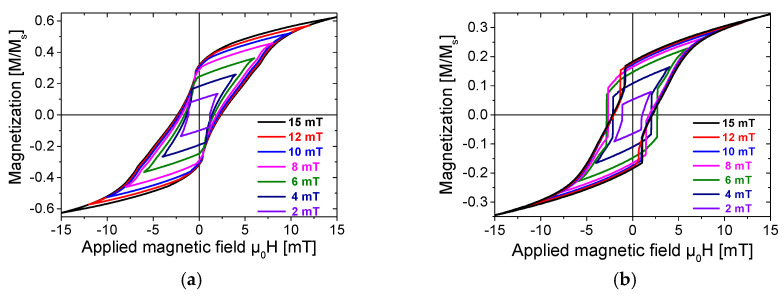
Magnetization curves of the Wiegand wire: (**a**) “only wire” model; (**b**) “affected wire” model.

The minor hysteresis loops of the Wiegand wire, measured under applied magnetic fields with different maximum amplitudes, are presented in Figure 3. Specifically, Figure 3a depicts the “only wire” model, while Figure 3b shows the “affected wire” model.

Notably, after attaching ferrite beads, the Wiegand wire demonstrates a more pronounced Barkhausen jump, with a significant increase in the jump amplitude of its magnetic induction intensity. This effect is particularly notable at $\mu_{0}H$ = 4 mT, 6 mT, and 8 mT. In fact, for the “only wire” model, no Wiegand effect was observed when the amplitude of the applied magnetic field was $\mu_{0}H$ = 2 mT, 12 mT, or 15 mT. In contrast, after adding ferrite beads, a distinct Wiegand effect was induced under these magnetic field amplitudes.

**Figure 4:**
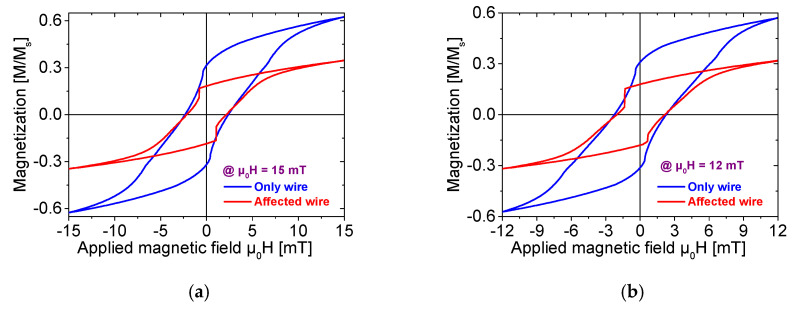

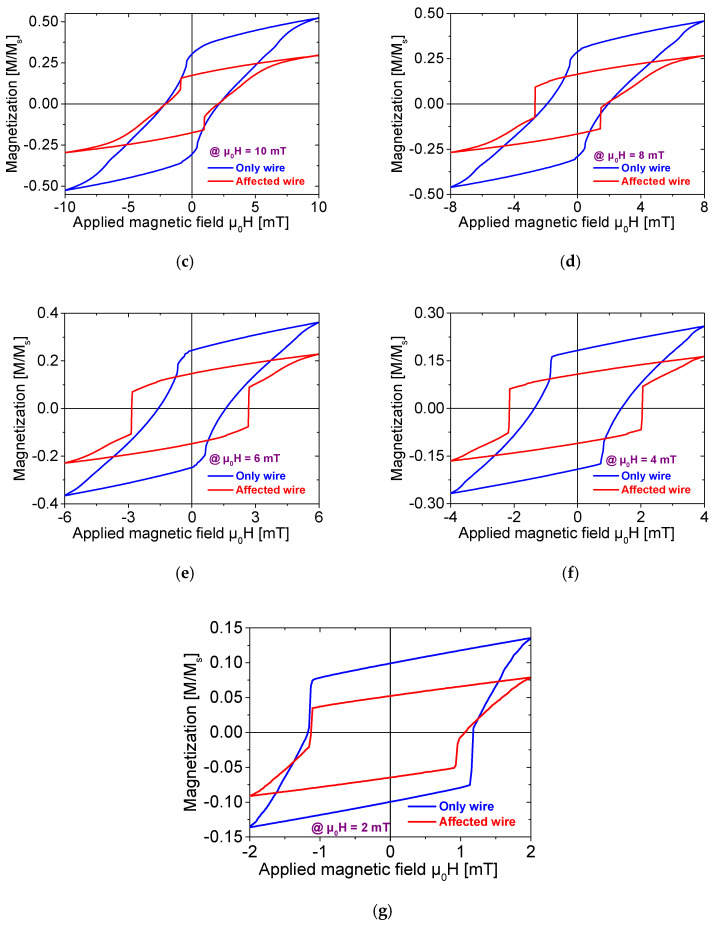
Minor hysteresis loops of Wiegand wires measured at applied field intensities of (**a**) $\mu_{0}H\;=\;15\;\text{mT}$; (**b**) $\mu_{0}H\;=\;12\;\text{mT}$; (**c**) $\mu_{0}H\;=\;10\;\text{mT}$; (**d**) $\mu_{0}H\;=\;8\;\text{mT}$; (**e**) $\mu_{0}H\;=\;6\;\text{mT}$; (**f**) $\mu_{0}H\;=\;4\;\text{mT}$; (**g**) $\mu_{0}H\;=\;2\;\text{mT}$.

To more clearly compare the influence of ferrite beads on the Wiegand wire under different external magnetic field intensities, Figure 4 provides a direct comparison of hysteresis loops between the “affected wire” and “only wire” models at $\mu_{0}H$ = 2 mT, 4 mT, 6 mT, 8 mT, 10 mT, 12 mT, and 15 mT, respectively.

Consistent with the observations in Figure 2, under each applied magnetic field intensity, the residual magnetic induction intensity of the Wiegand wire with ferrite beads shows a significant reduction. This reduction results in a significant reduction in the rate at which the magnetization curve reverts to the saturation magnetic induction intensity, ultimately resulting in a notable decline in the saturation magnetic induction intensity itself.

Furthermore, the Barkhausen jump of the “affected wire” model demonstrates is particularly prominent, with its jump amplitude greatly enhanced. Notably, at $\mu_{0}H$ = 4 mT, 6 mT, and 8 mT (i.e., Figure 4d–f), the Barkhausen jump in the “affected wire” model is accompanied by an increase in the coercive field. At the same time, the magnetization intensity of the wire can instantaneously switch from negative to positive, which results in a substantial increase in magnetic permeability and thereby effectively enhances the energy of the Wiegand pulse.

**Figure 5:**
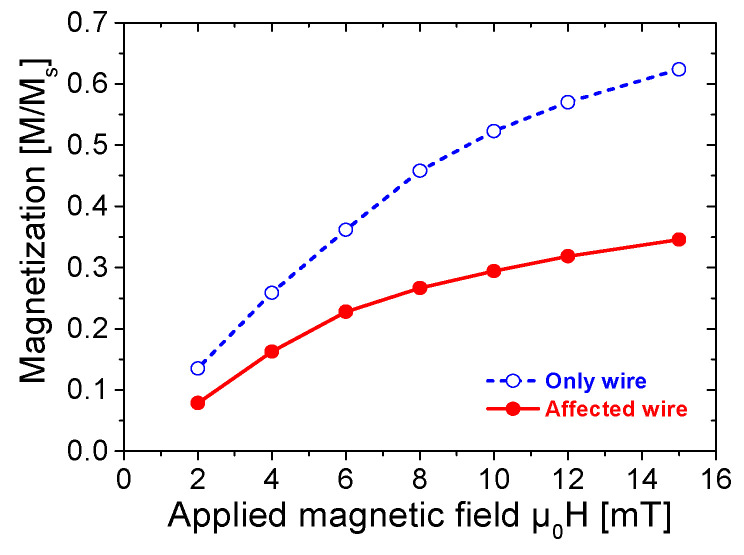
Magnetization intensity variation as a function of applied field, measured at room temperature (25 °C).

Figure 5 demonstrates that, with the increase in the external magnetic field, the magnetization rate of the Wiegand wire with the ferrite beads attachment decreases significantly.

### 3.2. First-Order Reversal Curves

FORCs were acquired after magnetizing the sample to saturation to reset its magnetic history. In this work, the FORCs were measured under an applied magnetic field ranging from $\mu_{0}H_{a}$ = −500 mT to $\mu_{0}H_{\mathrm{sat}}$ = 500 mT. Several open-source software programs, such as FORCinel (version 3.08) [22] and VARIFORC (version 4.01) [23], enable the calculation of FORC distributions and plotting FORC diagrams. Advanced functions of the VARIFORC are accessible from the FORCinel menu. Therefore, FORCinel and its auxiliary software (Igor Pro^®^, WaveMetrics Inc., Portland, OR, USA) were employed for data processing in this study.

**Figure 6:**
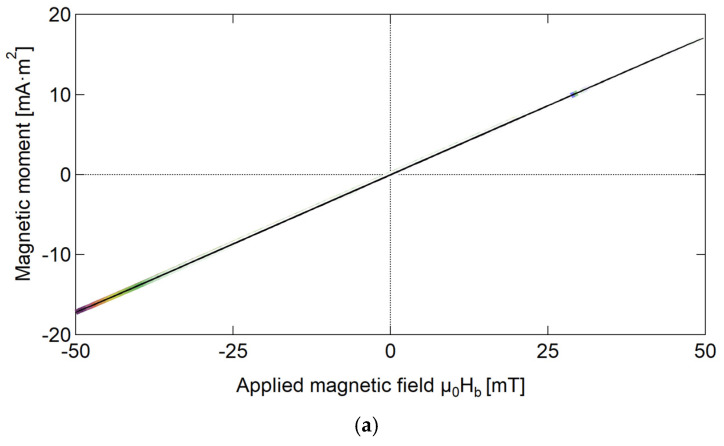

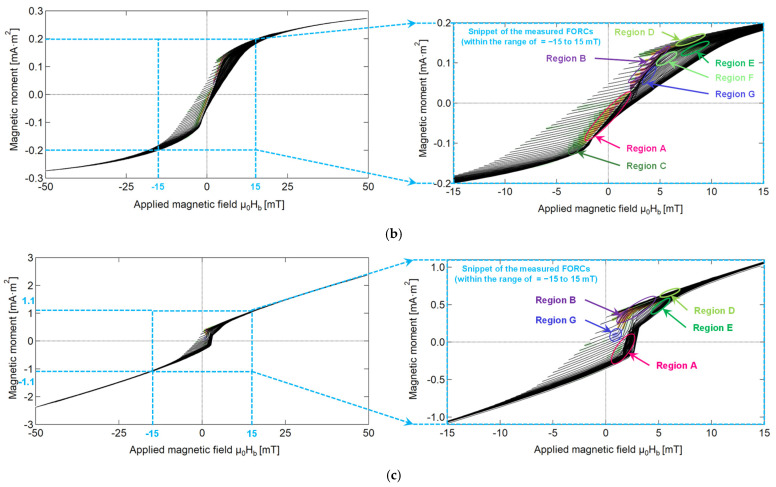
First-order reversal curves (FORCs) of Wiegand wire measured under applied magnetic fields ranging from −500 to 500 mT: (**a**) “only beads” model; (**b**) “only wire” model; and (**c**) “affected wire” model.

Each FORC was obtained by adjusting the sample’s magnetization through varying $\mu_{0}H_{a}$ in increments of 0.5 mT. Figure 6a–c illustrate the segments of the measured FORCs (within the range of $\mu_{0}H_{a}$ = −50 to 50 mT) and the snippet (within the range of = −15 to 15 mT) corresponding to the “only beads” case, the “affected wire” model, and the “only wire” model.

**Figure 7:**
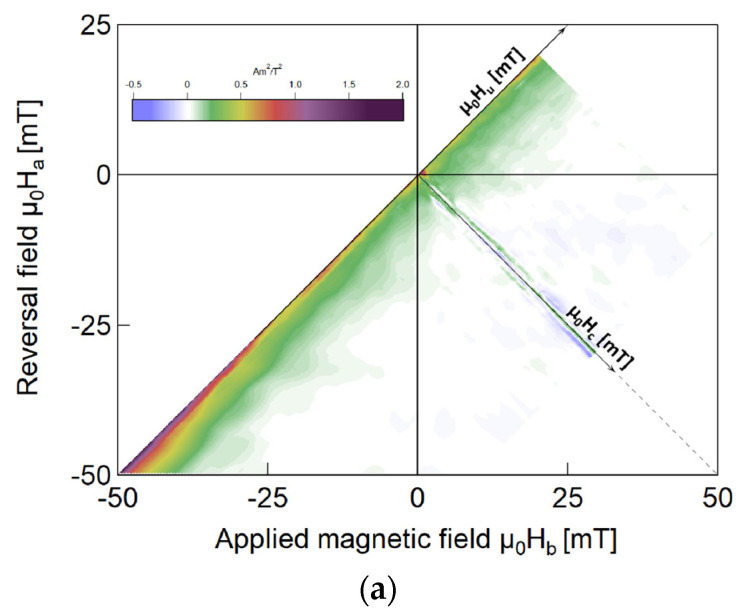

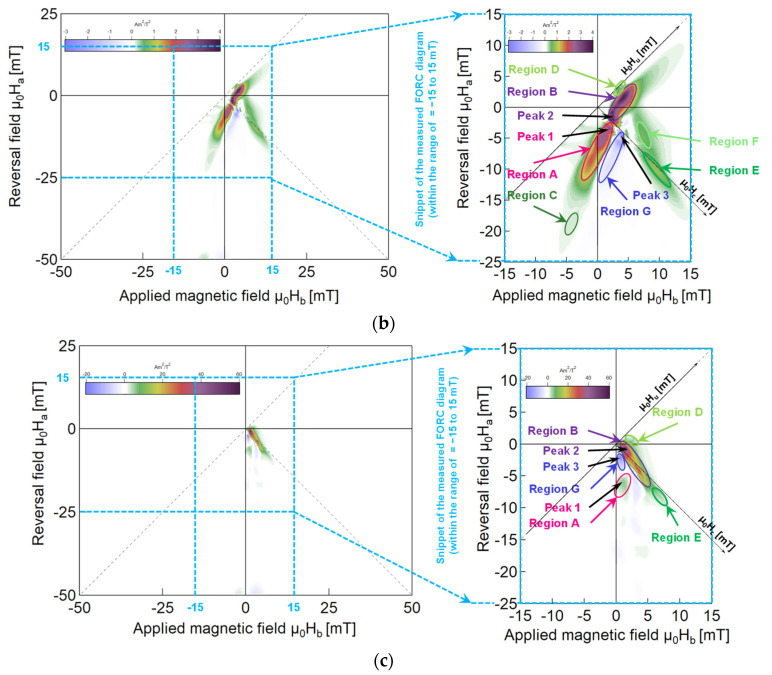
FORC diagrams of the Wiegand wire measured under the applied magnetic fields ranging from −500 to 500 mT: (**a**) “only beads” model; (**b**) “only wire” model; and (**c**) “affected wire” model.

Figure 7a–c show the FORC diagrams for the “only beads” case, the “affected wire” model, and the “only wire” model. In the FORCinel software, the FORC processing parameters were as follows: central ridge (Sb0) and vertical ridge (Sc0) are 4 and 3, respectively; vertical smooth (Sb1) and horizontal smooth (Sc1) are both 7; horizontal lambda and vertical lambda are both 0.1; the output grid is 1; and the central ridge vertical offset is 0.

## 4. Discussion

### 4.1. Enhancement of Irreversible Magnetization

As demonstrated in Figure 4a–f, compared with the “only wire” model, the primary effect of adding ferrite beads is a significant increase in the amplitude of the Barkhausen jump. Notably, the “affected wire” model demonstrates more distinct discontinuous transitions during magnetization reversal, with a well-defined critical field range.

Taking the hysteresis loop at a reversal magnetic field of $\mu_{0}H$ = 6 mT as an example, without ferrite beads, the magnetization reversal process occurs gradually with no clear abrupt critical point. In contrast, after attaching ferrite beads, a distinct “step-like” transition is observed when the external magnetic field is $\mu_{0}H$ ≈ 3 mT. This phenomenon indicates that ferrite beads modify the magnetization rate of the Wiegand wire. When the applied external magnetic field reaches the coercive field, the magnetization rate undergoes rapid enhancement and even reverses, resulting in a large-amplitude Barkhausen jump.

This enhancement of magnetization is particularly pronounced in the comparisons at $\mu_{0}H$ = 4 mT, 6 mT, and 8 mT, where Barkhausen jump amplitudes increase by 200–300%. The large Barkhausen jumps predominantly originate from the soft magnetic layer of the Wiegand wire. The results demonstrate that the “affected wire” model exhibits magnetization in a larger volume of the soft layer than the “only wire” model, and the magnetization volume of the reversed soft layer is proportional to the amplitude and energy of the output Wiegand pulse [8], ultimately yielding 1.5–2.0 times higher pulse amplitudes and a 30–40% greater total energy output.

The installation of ferrite beads transforms the interaction layer between the soft layer ($\mu_{0}H$ = 2 mT) and the hard core ($\mu_{0}H$ = 8 mT) into a soft layer, thereby increasing the volume of the reversed soft layer in the Wiegand wire. The magnetization reversal of the soft layer in the Wiegand wire exhibits irreversible characteristics [16]. In other words, installing ferrite beads can increase the irreversible magnetization of the Wiegand wire.

### 4.2. Transformation of Magnetic Structure

FORC analysis has become increasingly prevalent in research on magnetic materials as an advanced hysteresis measurement method. This is because it enables the acquisition of additional information to characterize magnetic properties, which are unattainable using normal hysteresis curves [19,24]. FORC diagrams offer the comprehensive characterization of the internal magnetization processes and magnetic interactions of samples in the “only beads”, “only wire”, and “affected wire” models. As shown in Figure 6a, in the “only beads” case, the applied magnetic field induces complete magnetization in the ferrite beads. As the external magnetic field increases, the magnetization intensity changes uniformly, and its FORC diagram has a ridge on the $\mu_{0}H_{u}$ axis (i.e., at $\mu_{0}H_{c}$ = 0), as shown in Figure 4. This ridge is called a reversible ridge [25], which also indicates that the magnetization process of the “only beads” case is reversible.

In the “only wire” model, the A region (the soft layer of the Wiegad wire) is further away from the reversible ridge after adding ferrite beads. Its distribution is only evident when the applied external field $\mu_{0}H_{b}$ > 0, and its intensity is approximately 10 times that of the “only wire” model, further proving that the magnetization of the soft layer is irreversible and improved. Meanwhile, Region B (the interaction layer of the Wiegad wire) in the “only wire” model is an almost reversible magnetization near the reversible magnetization ridge. After installing ferrite beads, the reversible magnetization transitions from a broad distribution along the $\mu_{0}H_{u}$ axis to the irreversible magnetization with a diffused distribution along the $\mu_{0}H_{c}$ axis.

The reversal field corresponding to the new Region B ranges from $\mu_{0}H_{a}$ = −8 mT to $\mu_{0}H_{a}$ = 1 mT, and the applied external field spans $\mu_{0}H_{b}$ = 1 mT to $\mu_{0}H_{b}$ = 6 mT. The magnetic field range in this area aligns with the applied alternating magnetic field, where a particularly significant Wiegand effect is observed (as shown in Figure 4). This further proves the assertion in Section 4.1 that the interaction layer transforms into a soft layer. This results in a “soft layer” with a coercive force of approximately $\mu_{0}H$ = 2 to 8 mT and accompanied by significant, large Barkhausen jumps.

The C and F regions, which were originally associated with irreversible magnetization in the A region (soft magnetic layer) and E region (hard magnetic core), exhibit a significant reduction in the “affected wire” model. This phenomenon may result from the irreversible magnetization enhancement induced by magnetic beads, resulting in C and F merging into their respective A and E regions. In future research, a quantitative analysis will be conducted to clarify this observation.

In both the “only wire” model and “affected wire” model, Region D belongs to the interaction layer and is distributed near the reversible ridge, exhibiting nearly reversible magnetization characteristics. However, in the “only wire” model, a distinct B region persists in the interaction layer, with the D region adjacent to the B region. Large Barkhausen jumps are evident in the B region of the “affected wire” model, indicating a transition from the interaction layer to the soft layer. Region E appears in the range of $\mu_{0}H_{b}$ > 5 mT and $\mu_{0}H_{a}$ < −8 mT. The $\mu_{0}H_{b}$ and $\mu_{0}H_{a}$ values in this region are far greater than the coercive field of the soft layer, indicating that it corresponds to the irreversible magnetization reversal process of the hard core.

### 4.3. Modification of Interaction Distributions

The B region undergoes a transition from the interaction layer (exhibiting nearly reversible magnetization) of the original “only wire” model to the soft layer (demonstrating irreversible magnetization) of the “affected wire” model. This change in the interaction distribution within the Wiegand wire was experimentally confirmed. These findings further support the assertion made in Section 4.1 regarding the enhancement of irreversible magnetization. They also demonstrate that the ferrite beads significantly alter the interaction distribution of the Wiegand wire.

Notably, although the marker colors of the two models are the same, their scales differ substantially. The distribution value of the “affected wire” model exhibits a substantial increase, with the peak value of the new Region B being approximately 10–15 times that of the original Region B, further confirming the stronger irreversible magnetization reversal effect caused by the ferrite beads.

Figure 7c reveals that after adding ferrite beads, a negative region (Region G) still persists in the FORC diagram, located at approximately $\mu_{0}H_{b}$ = 1 mT and $\mu_{0}H_{a}$ = −3 mT. In a previous study [16], a detailed analysis was performed of the reasons for the negative region explained by scholars such as Muxworthy and Carvallo, concluding that the negative region of the Wiegand wire arises from magnetic interactions. Reference [25] clearly states that the negative region originates from the coupling of reversible magnetization and irreversible state in the system. These results demonstrate that the negative region of the Wiegand wire stems from the coupling between the reversible magnetization component (interaction layer) and the irreversible magnetization components (soft layer and hard core). Furthermore, the presence of a negative region can be exclusively attributed to the curvilinear hysteron [25]. This finding further confirms that the magnetization process of the negative region in the FORC diagram is irreversible.

## 5. Conclusions

In this study, Wiegand wires composed of Fe_0.4_Co_0.5_V_0.1_ were used as the research object, with ferrite beads attached to both ends. Combined with the hysteresis loops (major and minor loops) and FORCs measured by a VSM, a systematic analysis was performed to elucidate the mechanism through which ferrite beads enhance the output performance of Wiegand sensors. The experimental results demonstrate that the integration of ferrite beads fundamentally modifies the magnetic properties of Wiegand wires via three synergistic mechanisms: (1) A significant expansion in the irreversible magnetization volume of the soft magnetic layer, quantified by a 10–15-fold increase in the intensity of FORC distributions and a 200–300% amplification of Barkhausen jumps within an external magnetic field range of 4–8 mT; (2) a portion of the interaction layer is transformed into the soft magnetic layer, and the coercive field of the soft layer shifts from 2 mT to 2–8 mT. The magnetization process of the interaction layer remains reversible, whereas the magnetization of the soft magnetic layer and hard magnetic core is irreversible; and (3) Region B has shifted from being distributed along the $\mu_{0}H_{u}$ axis (i.e., at $\mu_{0}H_{c}$ = 0) to being distributed along the $\mu_{0}H_{c}$ axis (i.e., at $\mu_{0}H_{u}$ = 0), leading to a modification in the interaction distribution. Additionally, the magnetization process of Region B has transitioned from almost reversible magnetization to irreversible magnetization. These structural and magnetic modifications collectively enhance the energy conversion efficiency by 30–40%, thereby establishing a comprehensive micromagnetic framework for the development of high-performance pulse-type energy harvesters in Internet of Things (IoT) systems. However, different types of ferrite beads exhibit distinct effects. In future work, comparative studies of various ferrite beads will be performed to maximize the energy output of Wiegand sensors.

## Data Availability

The original contributions presented in the study are included in the article. Further inquiries can be directed to the corresponding author.
